# Application of temperature, water stress, CO_2_ in rice growth models

**DOI:** 10.1186/1939-8433-5-10

**Published:** 2012-05-03

**Authors:** Jaeil Cho, Taikan Oki

**Affiliations:** 1 Department of Mechanical and Environmental Informatics, Tokyo Institute of Technology, 2-12-1 O-okayama, Meguro-ku, Tokyo, 152-8552 Japan; 2 Institute of Industrial Science, The University of Tokyo, 4-6-1 Komaba, Meguro-ku, Tokyo, 153-8505 Japan

**Keywords:** Temperature, Carbon dioxide, Water stress, Rice growth model, Review

## Abstract

In recent decades, numerous studies have attempted to project the impact of hypothesised anthropogenic climate change on rice production. In this study, we offer a comprehensive review of our current understanding related to temperature, CO_2_, and water-demand parameters in rice growth models. As to future rice yield, night time temperature should be focused in the models as well as day time temperature owing to the contribution of temperature on the night time respiration. Furthermore, although CO_2_-enhanced photosynthesis is critical for the accurate prediction of rice production in a higher CO_2_ atmosphere, we found that recent well-developed photosynthesis-stomatal model cannot realize the variation of CO_2_ stomatal sensitivity with humidity conditions. To estimate water stress under projected climate-change conditions, rice growth model should be required to link with water resource model, which includes natural processes and anthropogenic regulations. The understanding of abilities and limitations in the models is important not only to improve the schemes that models employ, but to also critically review the simulated results.

**Electronic supplementary material:**

The online version of this article (doi:10.1186/1939-8433-5-10) contains supplementary material, which is available to authorized users.

## Introduction

Rice is the most important staple food for a large part of the world’s population, especially in East and South Asia, the Middle East, Latin America, and the West Indies ([FAO, 2005]). As the population increases rapidly in these regions ([Coats, 2003]; [Bloom, 2011]), the demand for rice will grow to an estimated 2000 million metric tons by 2030 ([FAO, 2002]). To supply to this increasing demand, the methods of rice production will require significant improvement ([Ainsworth, 2008]). Achieving this goal, however, is sure to be a challenge with respect to future climatic changes ([Matthews et al., 1997]), which will basically be characterized by current global warming trends (Fischer et al., [2005]). The rise in temperatures and levels of carbon dioxide and uncertain rainfall associated with climate change may have serious adverse effects either directly or indirectly on the growth, development, and yield of rice crops ([Lobell et al., 2011]).

In recent decades, numerous studies have attempted to project the impact of hypothesised anthropogenic climate change on rice production ([Long et al., 2005, 2006]; [Tubiello et al., 2007]; [Ziska and Bunce, 2007]). Rice growth models are routinely used for assessing the impact of diverse agro-environmental changes on rice growth and yield ([Spitters et al., 1989]). Statistical models (e.g., [Kandiannan et al., 2002]; [Lobell and Burke, 2008]; [Auffhammer et al., 2012]) based on multiple regression analysis of historical yields and weather data are useful tools to estimate the impacts of climate trends upon rice yields. However, it becomes difficult to use the statistical models to examine the direct or indirect impacts of climate change in the good management of a rice paddy field ([Wang et al., 1991]), because the data reflected in the model contains the human effort being made to mitigate environmental shocks (negative effects) in order to maintain a high level of production. In addition, statistical models do not fully account for the physiological responses of rice to the unexpected and not-yet-experienced agri-environments that will result from dramatic climate change ([Lobell and Burke, 2008]). The results obtained will, thus, not enable us to fully understand how climate change will affect future food availability.

Increasing concern about the sustainable management of environmental resources and the effects of climate change on rice production has triggered the development of a number of sophisticated models based on physical and physiological processes ([van Laar et al., 1992]; [de Vries FWT et al. 1989]; [Kropff et al. 1993]; [Matthews and Hunt, 1994]). Process-based rice growth models of varying degrees of complexity can benefit from a comprehensive assessment of the response to likely climate changes ([Lansigan et al., 1997]; Bouman and Tuong, [2001]). As part of an effort to mimic a complex agrosystem, knowledge from experiments in both the field and laboratory will be incorporated into process-based models, using novel parameters and improved schemes. However, although these models are currently the best method available ([Bouman and Tuong, 2001]), they still rely on imperfect mechanistic processes and weak assumptions. Furthermore, these problems will not be solved even if a model includes all the parameters selected for an agricultural system, because the systems we are modelling are extremely complex. However, if we can demonstrate that certain parameters and schemes in a model are insignificant, then we can omit them to create a better and simpler model for good performance. Therefore, it is critically important to know which parameters will be the most significant for estimating rice productivity in the future environment.

With the current situation of global warming under the human-induced climate change, knowledge of the effects of (1) temperature, (2) CO_2_, and (3) water demand on the growth and development of rice crops has become essential over the past few years. In this study, we offer a comprehensive review of our current understanding related to temperature, CO_2_, and water-demand parameters. Our expectation is that this will be of significant use in understanding the development of the models for the prediction of future situations.

### Effects of temperature on rice yield

The impact of air temperature on rice growth would be location-specific because of the different sensitivity of different locations with regard to temperature. In tropical regions, the temperature increase due to the climate change is probably near or above the optimum temperature range for the physiological activities of rice ([Hogan et al., 1991]; [Baker et al. 1992]). Such warming will thus reduce rice growth. In addition, higher temperatures will cause spikelet sterility owing to heat injury during panicle emergence ([Satake and Yoshida, 1978]). In temperate regions, increased air temperatures should hasten rice development, thereby shortening the time from transplanting (or direct seeding) to harvesting and reducing the total time for photosynthesis yield development ([Neue and Sass, 1994]). Similarly, in high latitude regions, atmospheric warming may also increase the duration of the rice growing season. Therefore, a location-specific-parameterized rice model is not appropriate for modelling future environments globally ([Shimono et al., 2008]).

Although air temperature has conventionally been considered in the physiological processes of rice, the parameter of leaf temperature is more significant from the perspective of the energy balance on the leaf level, photosynthesis, and transpiration ([Morison and Gifford 1984]; [Lasseigne et al., 2007]). Therefore, the differences between leaf temperature and air temperature can create a significant uncertainty with regard to the season length and yield ([Baker et al., 1990]). For example, leaf temperatures could be warmer than the air owing to soil-surface influences, particularly in humid regions, which would result in a more rapid yield than that by air temperatures. The difference of 1°C will cause the change of leaf respiration by as much as approximately 1% of gross photosynthesis ([Mebrahtu et al., 1991]). However, only a few models calculate leaf temperature separately from air temperature, owing to the difficulty of parameterization of the related environmental factors. Basically, leaf temperature corresponds to air temperature ([Harley et al., 1985]). In addition, the temperature of an illuminated leaf is elevated by an amount proportional to the ratio of the incident radiation to a convention coefficient (e.g., humidity, wind speed, etc.) ([O’Toole and Tomar, 1982]). Therefore, a model requires the estimation of leaf temperature, using relevant mechanistic processes, in order to simulate the response of rice to changes in climatic variables.

Recent observation of climate variability shows an increase in the global mean surface air temperature, and a decrease in the diurnal temperature range ([Easterling et al., 1997]; [Braganza et al., 2004]; [Makowski et al., 2008]). This conclusion is derived from the fact that the daily minimum temperature is increasing at a faster rate than the daily maximum owing to the large specific heat of water, particularly in rice-growing areas ([Welcha et al. 2010]). Accordingly, in recent rice studies, attention has been directed to the effect of the daily minimum (night time) temperature. [Peng et al. (2004]), using field experiment data from 1979 to 2003, showed that yield might be more sensitive to the daily minimum temperature than to the daily maximum. The negative relationship between rice yield and daily minimum temperature is derived from the elevated specific dark respiration that takes place during the night time.

Indeed, the reported yields of maize, wheat, and soybeans cannot be fully understood by the relation of respiration to increased night time temperature ([Peng et al., 2004]). Nevertheless, the yield might be explained by their acclimation of crop dark respiration at higher temperatures, and the relationship between dark respiration and the previous daytime’s photosynthesis. The clear relationship between rice yield and daily minimum temperature implies that in a rice growth model, the physiological mechanisms of specific dark respiration are a priority in order to explain the effects of a warming atmosphere on the rice yield. Therefore, the simulation time-step should actually be on a less-than-daily scale, and the respiration scheme should be calculated separately from the estimation of the gross photosynthesis. However, models have mostly employed a daily time-step and have used a rather simple net calculation of the net photosynthesis, particularly for the estimation of future rice yield because the temperature projected by climate model still has sparse time scale. The possible overestimation of rice yield should thus be considered when discussing the simulated future yield.

### Interactive responses to increased CO_2_

Atmospheric CO_2_ has been increasing at a rather steady rate, at least on the time scale of a decade. During the last 50 years it has increased exponentially at a rate of approximately 2.4% per year. For this reason, there is continued interest in how rice will respond to future increases in CO_2_, since rice uses CO_2_ in its photosynthesis and growth. Most agronomic models use the magnitude of CO_2_ fertilization factor, which is mostly based on data from three literature reviews from the 1980s ([Kimball 1983]; [Cure and Acock 1986]; Allen et al. [1987]). According to laboratory experiments, rice grown at a higher CO_2_ level has more tillers than rice grown at an ambient level of CO_2_. Furthermore, the suppression of rice-specific dark respiration at high CO_2_ levels has been observed, despite the large variations in observed outputs ([Amthor 1991]; [Imai et al. 1992]; [Poorter et al. 1992]), and the physiological metabolism of nutrients in rice will become more critical under higher CO_2_ conditions.

According to previous enclosure experiments that have contributed to the physiological schemes used in rice growth models, the grain yield of rice will be promoted by higher CO_2_ levels. These studies, however, were probably conducted in the absence of other limiting factors. For example, [Long et al. (2006]) found that the photosynthesis stimulated in a rice free-air concentration enrichment (FACE) experiment is four times lower than the elevated CO_2_-enhanced value that is expected in enclosure studies. Given this circumstance, the most important scientific question is which physiological behaviours and environmental factors offset the direct fertilization effect of a rise in CO_2_ ([Harley et al., 1985]).

Indeed, positive performance under elevated CO_2_ would be directly associated with four key parameters: a decreased stomatal aperture, enhanced photosynthetic activity, increased total biomass, and changed biomass partitioning. The reduced stomatal aperture would produce an increased rice canopy temperature as a result of suppressed transpiration. It can thus mediate negative feedback in a warmed atmosphere. Despite a small stomatal aperture, CO_2_-enhanced photosynthesis will be produced by a rise in the intercellular CO_2_ concentration under a higher level of atmospheric CO_2_ ([Mott 1988]). However, following long-term subjection to a higher CO_2_ level, the net leaf photosynthetic rate of rice often declines from the expected value ([Imai and Murata 1978]; [Peet 1986]). This process is called the acclimation of rice photosynthesis to higher CO_2_. Although this acclimation is difficult to realize in a rice growth model because its mechanism remains unclear, the acclimation scheme of an effect that is in contrast to elevated CO_2_-enhanced photosynthesis might be critical for the accurate prediction of rice production in a higher CO_2_ atmosphere ([Makino et al., 1997]). In addition, considering the increased rice biomass that is produced in a higher CO_2_ atmosphere, which takes place through the increased photosynthesis and the decreased respiration, it is important for the process of biomass allocation to be accounted for in order to investigate these factors’ possible significance in a rice growth model. Previous measurement studies showed that specific leaf weight often increases in a higher CO_2_ atmosphere as a result of the thicker leaves and the increased number and length of the crown roots that are produced. However, the contribution of the changed leaf area or root biomass to the rice yield remains unclear.

**Figure 1:**
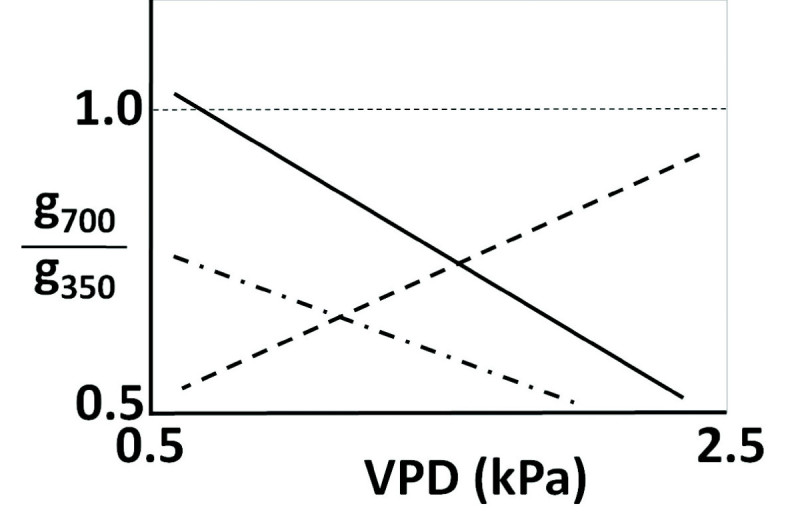
**Relationship between the ratio of leaf conductance at 700 μmol mol**^**-1**^**[CO**_**2**_**] to that at a 350 μmol mol**^**-1**^**[CO**_**2**_**] carbon dioxide concentration and the leaf-to-air-water-vapour pressure difference (VPD).** The solid line represents the measurement result of sorghum plants in [Bunce (2001]). The dashed line represents the results of the coupled photosynthesis-stomatal conductance model ([Collatz et al., 1991]). The dot-and-dashed line is based upon the measurement data for rice given in [Morison and Gifford 1983].

Although the response of rice growth to elevated CO_2_ is critically associated with other environmental factors, the interactive effects of simultaneous change have not been well investigated either in modelling studies or in experimental measurements. For example, few studies have mentioned the different sensitivity of stomatal behaviour to vapour pressure deficit (VPD) in terms of elevated atmospheric CO_2_ concentrations (Bunce 1998, 2001); [Katul et al. 2009]). Indeed, stomata, which are the strategic juncture for CO_2_ and water exchange between plant and atmosphere, are strongly linked not only to CO_2_ concentration, but also to the humidity as an atmospheric demand for moisture. The measurement data in [Bunce (1998, 2001]) shows that the ratio (g_s_700_/g_s_350_) of stomatal conductance at 700 μmol mol^-1^ to that at a concentration of 350 μmol mol^-1^ CO_2_ is negatively correlated with VPD to a significant degree (see Figure 1). However, according to our sensitivity test, one current well-developed model, which was proposed by [Collatz et al. (1991)] and which uses the biochemical photosynthesis model of [Farquhar et al. (19800] as well as the stomatal conductance model of ([Ball et al. 1987)], could not realize this actual behaviour (see Table 1 for formula). Basically, their coupled photosynthesis-stomatal conductance model can represent accurate stomata behaviours in response to CO_2_ and VPD under controlled conditions. Therefore, it is possible that failure of the model with regards to the interactive performance of CO_2_ and humidity can cause uncertainties as to the future rice yield and water demand, given the close linkage between the two processes in the two photosynthetic and stomatal schemes.

**Table 1:** **Summary of previous stomatal conductance (** ***g*** **) models**

No	Method reference	Equation	Forcing parameters	Coefficient	***Function***		
					***T***_***a***_	CO_2_	***H***
	***Oren-type (method by environmental factors)***						
1	[Oren et al. (1999])	g=−mln(VPD)+b	*VPD*	*m, b*	×	×	○
2	[Granier et al. (1996])	$g=[aR_{s}+b][1-m\ln(VPD)]$	*R*_*s*_ *, VPD*	*a, b, m*	×	×	○
3	[Lu et al. (2003])	$g=\{\begin{array}{l} aR_{s}+b\\ \left[R_{s}/(R_{s}+d)][a+b(c^{\mathrm{VPD}})\right] \end{array}$ $\begin{array}{l} if\;R_{s}<200\;W\;m^{-2}\\ if\;R_{s}>200\;W\;m^{-2} \end{array}$	*R*_*s*_ *, VPD*	*a, b, d, c*	×	×	○
	***Jarvis-type (method by environmental factors)***						
4	([Jarvis et al. 1976])	$g=g_{\max}f_{1}(PAR)f_{2}(VPD)f_{3}(T)f_{4}(S_{W})f_{5}(C_{a})$	*PAR, VPD, T, S*_*W*_ *, C*_*a*_	*g*_*max*_	○	○	○
5	[Massman & Kaufmann (1991])	$g=g_{\min}+g_{\max}f_{1}(PAR)f_{2}(VPD)f_{3}(T)f_{4}(S_{W})f_{5}(C_{a})$	*PAR, VPD, T, S*_*W*_ *, C*_*a*_	*g*_*max*_ *, g*_*min*_	○	○	○
6	[Noilhan and Planton (1989])	$g=g_{\min}LAI[f_{1}(PAR)f_{2}(VPD)f_{3}(T)f_{4}(S_{W})f_{5}(C_{a})]$	*PAR, VPD, T, S*_*W*_ *, C*_*a*_ *, LAI*	*g*_*min*_	○	○	○
7	[Avissar and Pielke (1991])	$\begin{array}{l} g=Ωg_{\max}[g_{\min}+(g_{\max}-g_{\min})\\ ñf_{1}(PAR)f_{2}(VPD)f_{3}(T)f_{4}(S_{W})f_{5}(C_{a})] \end{array}$	*R*_*s*_ *, VPD, T, W, C*_*a*_	*g*_*max*_ *, g*_*min*_ *, Ω*	○	○	○
	***Norman-type (Physiological models)***						
8	([Norman et al. 1982])	$g=\frac{A_{n}}{\left(C_{a}-C_{i}\right)}$	*A*_*n*_ *, C*_*a*_ *, C*_*i*_	*.*	○*	○	○
9	([Jones, 1992])	$g=1.6\frac{A_{n}}{\left(C_{a}-C_{i}\right)}\frac{RT_{k}}{p}$	*A*_*n*_ *, C*_*a*_ *, C*_*i*_ *, p,*	*.*	○*	○	○
10	[Knorr (2000)]	$g=\left(\frac{1}{1+b_{e}VPD}\right)1.6\frac{A_{n}}{\left(C_{a}-C_{i}\right)}\frac{RT_{k}}{p}$	*A*_*n*_ *, C*_*a*_ *, C*_*i*_ *, p, VPD*	*b*_*e*_	○*	○	○
11	Kim & [Verma (1991)]	$g=\left(1-\frac{VPD}{b_{D}}\right)1.6\frac{A_{n}}{(C_{a}-C_{i})-\frac{1.37}{A_{n}g_{l}}}$	*A*_*n*_ *, C*_*a*_ *, C*_*i*_ *, p, VPD*	*b*_*D*_	○*	○	○
	***Ball-type (Physiological models)***						
12	([Ball et al. 1987])	$g=m\frac{A_{n}}{C_{s}}h_{s}+b$	*A*_*n*_ *, C*_*s*_ *, h*_*s*_	*m, b*	○*	○	○
13	[Leuning (1995])	$g=m\frac{A_{n}}{\left(C_{s}-\Gamma \right)}h_{s}+b$	*A*_*n*_ *, C*_*s*_ *, h*_*s*_	*m, b, Γ*	○*	○	○
14	In this study	$g={\left(k\;C_{i}\right)}^{\left[1-(C_{a}/C_{i})(VPD/VPD_{\max})\right]}\frac{A_{n}}{\left(C_{s}-\Gamma \right)}h_{s}+b$	*A*_*n*_ *, C*_*s*_ *, h*_*s*_ *, C*_*i*_	*k, b, C*_*a*_ *, VPD*_*max*_	○*	○	○

Note: *H*: humidity, g: stomatal conductance, *R*_*s*_: incoming solar radiation, *T*_*a*_; air temperature, VPD: vapor pressure deficit, *S*_*w*_: soil moisture, *C*_*a*_: atmospheric [CO_2_], *C*_*i*_: intercellular [CO_2_], LAI: leaf area index, *A*_*n*_: net photosynthesis, *g*_*max*_: maximum stomatal conductance, *g*_*min*_: minimum stomatal conductance, Ω: leaf age function, *p*: air pressure of the standard atmosphere, *b*_*e*_ is the coefficient according to soil water status, *Γ*: CO_2_ compensation point in photosynthesis. (○*: indirectly considered in formula, ○: directly considered, ×: not considered).

### Consideration of water stress and demand in models

Rice cultivation is suited to regions with high rainfall because it requires ample water. However, it is expected that the hydrological cycle accelerated by a warmed atmosphere will change the pattern of rainfall in these regions. Basically, rice is very sensitive to a reduction in soil moisture, and rice production consumes much more water than the production of other crops. Approximately 500 L of water is required to produce 1 kg of biomass ([Jodo et al. 1995]). This high degree of water consumption is related to diverse physiological processes in rice. The transpiration that is reduced under stressful water conditions will result in the suppression of nutrient uptake by the rice root system. The reductions in leaf expansion and in the photosynthetic rate that result from moderate water deficits are responsible for a reduction in dry matter production and grain yield ([Gifford 1979]). Furthermore, drought during the flowering stage causes spikelet sterility and yield losses, particularly in upland areas ([Ekanayake et al., 1993]). These direct effects of water stress on numerous metabolic and physiological processes in rice are relatively well established in models, mostly using optimal trends obtained from field or laboratory experiments.

The difference between leaf and air temperatures is commonly used as an indicator of rice water stress ([Long et al., 2006]), because leaf temperature is more strongly correlated with transpiration than with photosynthesis. This implies that a shortage of water produces two important physiological responses: in leaf temperature and in transpiration. Water stress in combination with a warmed atmosphere will increasingly limit rice production owing to a much higher leaf temperature ([Garrity and O’Toole, 1994]). In addition, although water stress will simply result in stomatal closure, as in the case of elevated CO_2_ levels, the effects and possible interaction of elevated CO_2_ and water stress on rice stomatal behaviour are a critical issue to be considered in the current context of climate change ([Turner et al., 1986]). For example, a complementary acclimatization of photosynthesis in water stressed rice growth under elevated CO_2_ conditions has been reported, despite an increased rate of net leaf photosynthesis under these same conditions ([Imai and Murata 1978]; [Makino et al., 1997]). Therefore, the calculated water stress parameter is linked to diverse other schemes in a rice growth model because the physiological processes under water stress are complex and may vary according to the presence or absence of other stresses. This means that model developers have to validate and evaluate their models’ performance with regards to the effect of water stress on rice growth under a variety of environmental changes. It is critically important for us to predict rice yield for unexpected climate events.

In fact, the change in yield caused by water shortage is difficult to validate because of limited observation data for water stress. The critical parameters for water stress, such as leaf water potential and leaf temperature, are rarely investigated by in-situ measurement. However, we could indirectly predict the effect of a water deficit on field yields through the relationship between evaporation (or transpiration) and yield. Such information could be available from numerous previous studies ([Simpson et al., 1992]; [Bouman and Tuong, 2001]; [Tuong et al., 2005]). This relationship may or may not be linear, in part because the fraction of evaporation that does not contribute to rice growth varies throughout the rice life cycle. In addition, water-use efficiency (yield or biomass divided by evaporation) is another relevant parameter in evaluating the water demand for rice growth. This factor could be affected by climate change through changes in the irrigation water demand for rice growth. For example, increased evapotranspiration (from the water body of the paddy field and rice stomata) in a warmer atmosphere will require a greater amount of water. On the contrary, a higher temperature will result in a reduced number of irrigation days, on account of the decreased rice growth period ([Simpson et al., 1992]; [Bouman and Tuong, 2001]). Furthermore, water-use efficiency will increase at higher CO_2_ concentration levels owing to the expected decrease in transpiration and increase in photosynthesis (if the leaf temperature is constant). Therefore, in a rice growth model, the water stress scheme should not be designed in isolation from other environmental changes, such as air temperature, and CO_2_ level.

These likely interactive phenomena will affect water resource planning and management of irrigation water demand. In addition, the availability of water for rice production is dependent not only on the precipitation and environmental factors related to evapotranspiration, but also on irrigation management ([Tuong et al., 2005]). Indeed, the efficiency of water use for grain production is higher in a saturated soil culture than in an unsaturated soil moisture condition. In most cases, rice production is associated with flood irrigation. Although this method is simple, it also requires sound planning and servicing of the water damming and channelling. Therefore, in a rice growth model, this has to be emphasized to account for the variations of water resources, not only in terms of climate variability but also in terms of anthropogenic effects. However, the realization of terrestrial water resources is not an easy task in the design of a model because of the complex interactions among urban, industrial, agricultural, and natural water requirements. Recently, [Pokhrel et al. (2012]) introduced an integrated model to estimate terrestrial global water resources. The model contains four different anthropogenic water regulation modules (a crop growth module, reservoir operation module, water withdrawal module, and environmental flow requirement module; see also [Hanasaki et al. 2008] for more details) that operate on the basis of a surface and sub-surface runoff process module ([Stieglitz et al. 1997]) a hydrological and biophysical exchange module ([Takata et al. 2003]), and a river routing module ([Oki and Sud 1998]). Although their model is not directly coordinated with a rice growth model, such modules related to water resource assessment are needed in order for us to better investigate the growth stage of a water-sensitive crops such as rice.

## Conclusion

**Figure 2:**
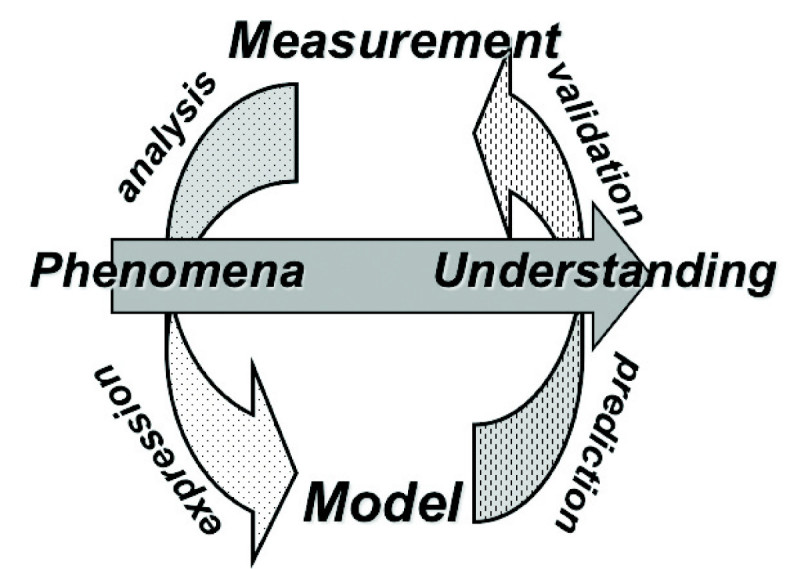
**Methods for including a measurement study and (rice growth) model study to understand a real phenomenon or anticipate a future situation.** The measurement and the model are generally linked through the approaches of analysis, expression, validation, and prediction.

Rice growth models are subject to many uncertainties. The conventional way of addressing this uncertainty has been to obtain comprehensive information about the physiological and phenological responses of rice to environmental factors. Even though a given model cannot take into account all the relevant processes, we need to critically understand which parameters will make a definite contribution to obtaining the desired result, and which will not. It is important not only to improve the schemes that models employ, but to also critically review the simulated results (see Figure 2). In this review, we have examined the existing or required processes related to temperature, CO_2_, and water stress in rice growth models because these are the three factors that are most vulnerable to climate change. It is thus obvious that we must understand the actual roles they play in the physiological processes of rice, as well as the ways these roles may be altered in order to predict rice yields in the future.

In addition, the interactive effects that result from both positive and negative factors are necessary if we are to consider uncertain climate changes. In this review, we have emphasized the importance of accounting for the interactive effects of temperature, CO_2_, and water demand in a model. However, although our knowledge of the effects that these three factors have on the growth and development of the rice has increased in the past few years, it has remained difficult to realize these interactive effects in a model because of unclear mechanisms involved and the limited experimental data. Therefore, theoretical considerations and experiments that are designed to increase our understanding of this issue are recommended, along with validation studies. ‘Semi-empirical schemes’ and ‘clear assumptions’ based on decades of agronomic knowledge might be the best approach, particularly in light of our limited understanding. Semi-empirical schemes derived from both mechanical approaches and observed characteristics will be suitable to reasonably reflect the latest validation results, particularly under projected climate-change conditions. Showing a clear assumption in a model will improve our understanding of a model’s results, and will also suggest further tasks to pursue in model development.

Fortunately, current rice growth models have been improving as a result of the kind of continuous validation and development mentioned in this review. These recent results for the projection of future rice yields will produce meaningful information that is of great use to society. However, the impact assessment related to global future food security could have a considerable bias owing to not only limitations of a model’s performance, but also to the uncertainties of future weather inputs ([Aggarwal, 1994]). For example, general circulation models (GCMs) still produce uncertain predictions as to how climate variability will vary as a consequence of an increase in greenhouse gases. Nevertheless, there is continued interest in how rice will respond to future changes in temperature, CO_2_, and water demand because climate change has been a consistent feature of the global climate. It is worth incorporating as the likely impacts of GCM-derived climate change scenarios into the results simulated in rice growth models’. Therefore, an accurate understanding of model behaviour and details is needed in order to produce accurate and well-organized information for our society.

## Authors’ original submitted files for images

Below are the links to the authors’ original submitted files for images. Authors’ original file for figure 1 Authors’ original file for figure 2
